# Usability, performance and safety of a new device for degenerative mitral regurgitation: *in vivo* chronic evaluation

**DOI:** 10.1093/icvts/ivac067

**Published:** 2022-03-15

**Authors:** Jacob Zeitani, Giovanni Alfonso Chiariello, Rona Shofti, Piergiorgio Bruno, Massimo Massetti, Ottavio Alfieri

**Affiliations:** 1 Neurosciences and Rehabilitation Department, University of Ferrara, Ferrara, Italy; 2 Cardiovascular Sciences Department, Agostino Gemelli Foundation Polyclinic IRCCS, Rome, Italy; 3 Catholic University of The Sacred Heart, Rome, Italy; 4 Department of Biomedical Engineering, Technion-Israel Institute of Technology, Haifa, Israel; 5 Department of Cardiac Surgery, San Raffaele University Hospital, Milan, Italy

**Keywords:** Mitral regurgitation, Mitral valve repair, Mitral ring, Chordae implantation

## Abstract

**OBJECTIVES:**

This study aimed to evaluate the usability, performance and safety of an innovative mitral valve device in the chronic setting characterized by an intraventricular bridge, which enables artificial chordae anchoring and/or direct posterior leaflet fixation.

**METHODS:**

Ten female sheep were employed and underwent device implantation. Any interference of the device with leaflet motion, ease of device use, correct chordae length estimation and implantation were evaluated. Post-procedural valve competence and device performance were verified by periodic postoperative echocardiograms and laboratory examinations. Following euthanasia, gross anatomy and histology evaluation of the hearts and valves were performed to detect tissue abnormalities and inflammation reaction related to the device.

**RESULTS:**

The procedure was successfully completed in all 10 sheep. Lengths of the 2 chordae implanted were 23 (21.5–24) mm and 23 (22.5–24) mm. The time required to suture both pairs of the artificial chordae was 2.7 ± 0.7 min. At the 3-month follow-up, left ventricular function was normal. The transvalvular peak pressure gradient was 9 (7.5–10) and the mean gradient was 4 (3.5–4) mmHg. Upon necropsy and histological evaluation, no damage to left ventricle wall, valve leaflets, chordae and papillary muscles and absence of thrombus formation and inflammatory reaction were observed. Radiological images showed neither fracture of the device nor calcifications. Laboratory tests showed no signs of haemolysis.

**CONCLUSIONS:**

*In vivo* late tests confirmed the ease of correct chordal length estimation prior to implantation, short operative time and usability in flailed anterior leaflet repair. The absence of negative impact of the device on mitral leaflets motion, function and structure and successful repair might suggest that the device would be useful in complex degenerative mitral disease.

**Figure:**
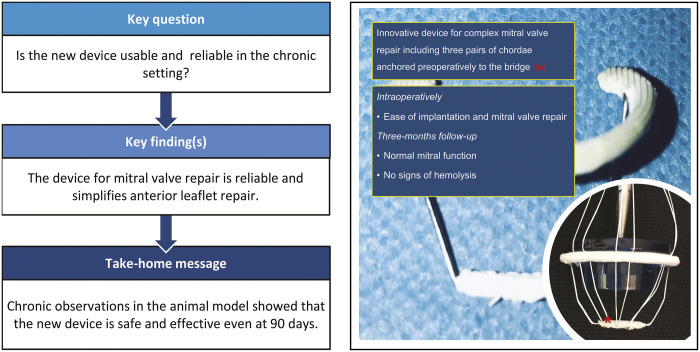

## INTRODUCTION

Although mitral valve repair (MVR) is generally considered superior to valve replacement for the treatment of degenerative mitral regurgitation (MR), the repair rate still remains low in many cardiac surgery centres. Several factors might influence the surgeon’s decision, including low volume mitral valve procedures, valve morphology, particularly when both leaflets are involved or complex anatomy, with a relevant risk of systolic anterior motion, resulting in a higher recurrence of MR rate [1–9]. Long-term results may be associated with the correct or perfect repair of valve insufficiency during surgery and late left ventricle (LV) remodelling following repair [10–12]. Besides annular shape, depth of leaflets coaptation may also contribute to achieving durable results of surgery. Thus, the progression of degenerative disease with chordae elongation and left ventricular remodelling may be compensated by adequate leaflet coaptation depth, which keeps the valve competent.

In most repair procedures, a prosthetic ring is usually implanted to guarantee annular stability and durability of result. However, currently available rings are intended solely to treat the annular deformity and are used to achieve better leaflets coaptation by forceful under or over sizing, regardless their shape and grade of rigidity, which increases the risk of suture dehiscence and device fracture [13]. The innovative device that we proposed and already presented in its initial *in vitro* and *ex vivo* tests is characterized by an annular ring portion and by an intraventricular segment, thus presenting a three-dimensional configuration for not only annulus reshaping and stabilizing but also for leaflet anchoring directly or by means of artificial chordae [14]. The device is conceived to overcome technical complexities of chordal length estimation, papillary muscle exposure and suturing and to simplify complex repair and late LV remodelling-related volume changes and consequent mitral incompetence [11, 15, 16].

In previous *in vitro* and *ex vivo* studies, the device proved to be very effective in mitral insufficiency repairing by means of chordae implantation. Fatigue and functional analyses showed that the device apparently is safe and durable, and its structure does not interfere with valve function, with normal movements and coaptation of the leaflets during cardiac cycles. Preliminary acute *in vivo* tests in sheep confirmed the easy pre-anchoring of the chordae to the device bridge and preoperative length estimation, resulting in short operative time. The device showed no negative impact on mitral leaflet motion and function and surrounding cardiac structures were detected.

The purpose of this 3-month chronic *in vivo* study was to confirm the ease of artificial chordae length estimation and anchoring before implantation, as in the previous test, and to evaluate any adverse events related to the device in a longer observation time, including valve and left ventricular performance, its haemolytic effect, tissue overgrowth, thrombi or valvular structural rupture or damage to the device.

## METHODS

### Ethics statement

The experimental protocol was approved by the Institutional Ethics and the Institutional Animal-Care and Use-Committee of Pre-Clinical Research Authority Technion, Haifa, Israel (protocol no. IL-032-03-2018). All animals received humane care in accordance with the 1996 ‘Guide for the care and use of laboratory animals’ recommended by the US National Institute of Health (NIH Publication No. 85-23, revised 1996).

### Device characteristics and utility

**Figure 1:**
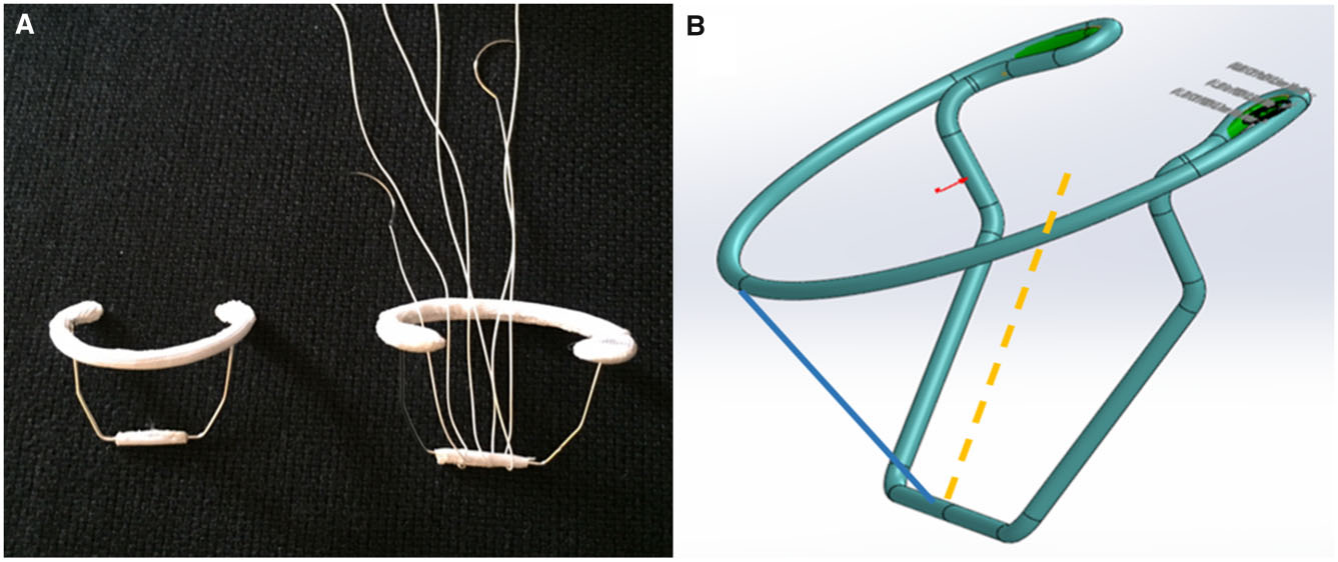
The innovative mitral valve device designed to allow anchoring of artificial chordae implanted for mitral valve repair (**A**). Representation showing the estimate of the chordal length before implantation. The length of the neochordae is calculated from the bridge to the ring plane. For posterior leaflet, a vertical line (blue line) and for the anterior an oblique line (orange dashed line) are measured (**B**).

The proposed device (InnerCore Medical, Ltd., Tel Aviv, Israel), made of cobalt chromo (Fort-Wayne-Metals CrCo 35NLT, CW50-60%), is composed of a C-shaped incomplete ring body, to be implanted on the posterior aspect of the mitral valve annulus. An anterior portion is connected to 2 legs being configured for crossing through the valve commissures. The legs are interconnected via a bridge, designed to allow anchoring of artificial chordae and/or directly to 1 or both mitral leaflets (Fig. 1A). When artificial chordae are applied, they are anchored directly to the device bridge preoperatively rather than being sutured to the papillary muscles, as previously described. Briefly, the distance between the bridge and the ring is well known and the chordae length can be measured and signed. A vertical line for the posterior leaflet is used, whereas an oblique one is used if the anterior leaflet is involved (Fig. 1B). Numerous chordae can be anchored and used.

Device characteristics, its dimensions and manufacturing material have been previously described [14].

### Animal model

Asaf female sheep were selected and underwent device implantation. Animal selection criteria were normal heart and optimal valve function.

Considering the small study sample and the three-dimensional device configuration, we thought to use one size of the device, and so conditioning sheep selection with regard to mitral valve size. A nominal 24-mm (with external 27–28 mm swing cuff measured at the intercommisural segment) devices were produced, and sheep with mitral intercommisural distance between 30 and 34 mm were preferred, as assessed by preoperative transthoracic echocardiogram.

All animals underwent open heart surgery via left thoracotomy and cardiopulmonary bypass (CPB). Following the first procedures (6 sheep) where device-valve apparatus was assessed, the MV was made incompetent by severing the primary chordae of the anterior leaflet (A2) in the last 4 sheep and repaired using 2 pairs of 5–0 Gore-Tex artificial chordae. The device was lowered into the valve and sutured to the annulus by anchoring it to the fibrous trigones passing the stitch through the eyelet at the 2 extremities of the ring. MV competence was then assessed by cold saline injection in the ventricular cavity.

### Investigational protocol

The device was implanted in 10 female sheep (Table 1). At surgery, to generate mitral valve prolapse, in the last 4 sheep, primary anterior leaflet chordae (A2) were severed and replaced by 2 pairs of 5–0 Gore-Tex artificial chordae (W.L. Gore&Associates, Langstaff, AZ, USA), which were preoperatively anchored to the device bridge. The primary end points of this study were the impact of the device on the valve apparatus, anterior leaflet repair for prolapse, ease of artificial chordae length estimation, ease of subsequent chordal anchoring, mitral valve competence, mitral and surrounding tissues degeneration and/or overgrowth, blood and haemolysis investigation and device fracture or damage for up to 3 months of follow-up.

**Table 1:** Animal features and procedural technical characteristics in experimental device implantation

Characteristics	Value
Sheep number	10
Body weight, kg, median (25th–75th)	94 (87.5–97)
CPB time, min, median (25th–75th)	69 (62.7–73.5)
Aortic cross-clamping time, min, median (25th–75th)	35 (32.2–38)
Sutures needed for implant (*N*)	8–10
Neo-chorda 1 length, mm, median (25th–75th)	23 (21.5–24)
Neo-chorda 2 length, mm, median (25th–75th)	23 (22.5–24)
Time for chordal implant, min, median (25th–75th)	3 (2–3)

Median (25th–75th): median and interquartile range (25th–75th percentile).

CPB: cardiopulmonary bypass.

Before the operation, animals were acclimatized for 5 days for a general clinical check-up. Baseline laboratory tests were performed, including blood cell count, complete blood chemistry, prothrombin time, partial thromboplastin time, international normalized ratio, haematocrit (Hct), haemoglobin (Hb) level and lactic dehydrogenase (LDH) level. During follow-up, all animals were controlled and assessed daily by the veterinarian team for auscultation, general conditions and weight.

### Echocardiography evaluation

Preoperatively sheep were selected based on transthoracic echocardiogram (ACUSON SC2000 Ultrasound System-Siemens Healthineers, Erlangen, Germany). Intraoperatively epicardial echocardiogram was performed before CPB and at the end of surgery before chest closure. Transthoracic follow-up echocardiograms were performed on days 1, 7, 14, 30, 60 and 90 postoperatively. Notably, considering the unfavourable anatomy of the sheep, particularly the oesophagus, heart transoesophageal echocardiogram was not performed. All echocardiograms were performed by the same physician (Table 2 and Video 1).

**Table 2:** Postoperative echocardiographic data at 90-day follow-up

Echocardiographic data	Value
Surviving sheep, *n* (%)	7 (70)
LVEF (%), median (25th–75th)	60 (58.7–65)
Absence of mitral regurgitation, *n* (%)	7 (100)
Trans-mitral PPG. mmHg, median (25th–75th)	9 (7.5–10)
Trans-mitral MPG, mmHg, median (25th–75th)	4 (3.5–4)
Anterior leaflet motion, *n* (%)	
1	0 (0)
2	0 (0)
3	0 (0)
4	7 (100)
Posterior leaflet motion, *n* (%)	
1	0 (0)
2	0 (0)
3	0 (0)
4	7 (100)

Anterior/posterior leaflet motion: 1: absent; 2: mild; 3: moderate; complete : 4. Median (25th–75th): median and interquartile range (25th–75th percentile).

LVEF: left ventricular ejection fraction; MPG: mean pressure gradient; PPG: peak pressure gradient.

### Blood tests

Blood samples at baseline and in the follow-up period were obtained to evaluate organ function, Hb levels and possible laboratory markers of haemolysis, such as LDH, bilirubin, Hb and Hct.

### Surgical procedure

Animals were anaesthetized using intramuscular injection of ketamine (20 mg/kg) and of xylazine (0.01 mg/kg) and intravenous injection of propofol (5–7 mg/kg). Animals were then intubated with a cuffed endotracheal tube and ventilated with a volume-control Drager Fabius Tiro ventilator (Drager Medical GmbH, Germany). Fentanyl (5 mg/kg/h) was intravenously administered. Anaesthesia was maintained with isoflurane and propofol. Electrocardiogram, arterial blood pressure, O2 arterial saturation and rectal temperature values were continuously monitored. Immediately before surgery, 3 g of cefazolin antibiotic was intravenously administered.

**Figure 2:**
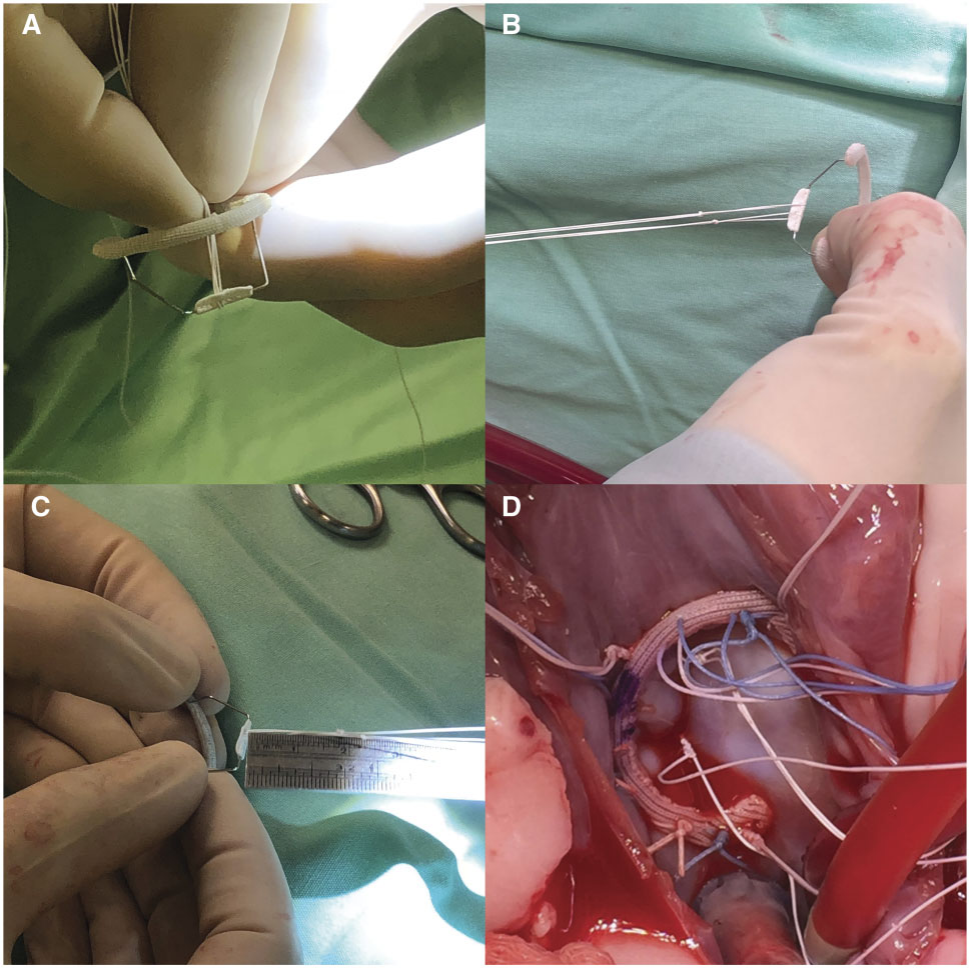
Artificial chordae anchoring on the interventricular bridge device before implantation (**A** and **B**). Measurement of chordal length (**C**). Device implantation (**D**).

After skin shaving and disinfection, the chest cavity was accessed through a left thoracotomy in the fifth intercostal space. The left lung was compressed by wet gauze and inflated continuously with 2 l/min of CO2. The pericardium was opened longitudinally anterior to the phrenic nerve, and stay sutures were placed in the pericardium to facilitate access to the left atrial appendage. Two-dimensional epicardial echocardiogram was performed at baseline. After the intravenous administration of heparin (300 U/kg) to achieve an activated clotting time of >350 s, the descending aorta (20-Fr cannula, Edwards Lifesciences, CA, USA) and right atrium (two-stage 36-Fr cannula, Medtronic, Inc, Minneapolis, MN, USA) were cannulated. CPB was initiated using a Terumo oxygenator (Terumo Corp., Terumo, Japan), lowering the body temperature to 32°C. The ascending aorta was cross-clamped proximally to the brachiocephalic trunk, and cold antegrade blood cardioplegia was delivered in the aortic root to achieve a diastolic cardiac arrest. Cardioplegia was repeated every 30 min. After a cardiac arrest, the left atrial appendage was opened, and the MV was exposed. Eight to ten 2–0 double-armed polyester sutures were placed in the posterior aspect of the mitral annulus up to the trigons. In all instances, a 24-mm ring was used. In the first 6 sheep, the valve remained intact, and the device was implanted. In the last 4 sheep, the primary chordae of A2 were torn and replaced by 2 pairs of artificial chordae, to evaluate the reliability of the ring in case of complex and challenging repair as the anterior leaflet. The artificial chordae were measured and anchored to the bridge of the device preoperatively, considering the distance of the bridge to the ring due to the required chordae length. When the artificial chordae were used, they were passed along the anterior leaflet free margin before lowering and fixing the device to the annulus and knotted as the last procedure. Valve competence was then assessed by cold saline injection in the ventricular cavity (Fig. 2).

The animals were rewarmed, the left atrial appendage was closed, the cardiac chambers were de-aired, and the aortic cross-clamp was removed. After weaning off CPB, the cannulae were removed and adequate reversal of heparinization with protamine was controlled by measuring the activated clotting time. Epicardial 2D echocardiogram was repeated to assess device position and valve function.

After chest closure, the sheep were awakened and extubated in the operative room. Blood pressure and vital parameters were followed for ∼1 h before transferring to the recovery area to continue the observation. During follow-up, all sheep were anticoagulated with subcutaneous injections of 6000 U of clexane every 12 h for 1 week. Clopidogrel 75 mg/die and acetylsalicylic acid 100 mg/die were started after chest drain removal on day 1 postoperatively. Antibiotic treatment with intramuscular administration of 20 mg/kg of cefuroxime was continued for 7 days. Tramadol (1000 mg per os every 12 h) and tolfenamic acid (4 mg/kg a day, intramuscular) were used as analgesics for 5 days.

### Long-term management

A general clinical check-up was performed daily. Blood tests for estimating serum bilirubin, Hb, Hct and LDH dosage were performed during the period of observation until euthanasia (blood tests were taken preoperatively and at days 0, 1, 2, 7, 14, 30, 60 and 90 postoperatively). Transthoracic echocardiograms were performed on days 1, 7, 15, 30, 60 and 90 postoperatively, also considering mitral leaflets motion with a range of values between 1 (no leaflet motion absent) and 4 (normal leaflet motion) (Table 2 and Video 1).

### Study termination

All surviving animals (7 out of 10) were euthanized at 3 months postoperatively. In surviving animals, the hearts were then explanted for direct inspection, immersed and fixed in 10% neutral buffered formalin and evaluated by Alizée Pathology, Inc. (Thurmont, MD, USA).

The MV and the device were examined for problems such as structural fracture or damage, tissue overgrowth on the ring and its intraventricular bridge portion, thrombi or vegetations. Particular attention was paid to the dehiscence of the device and any surface abnormalities as well as any possible downstream changes in the myocardium.

### Gross anatomy and histology

According to the study protocol, animals were subjected to necropsy at the end of a 3-month period, consisting of an examination of the heart and the test device. The hearts were harvested and immersed and fixed in a 10% neutral buffered formalin.

Whole hearts and explanted mitral valve were radiographed at 2 orthogonal views,

Hearts were trimmed above and below the mitral annulus to expose the test device. Following the gross assessment, the tissues were dehydrated, infiltrated and embedded in a Spurr resin. The resulting plastic (Spurr) blocks were photographed and sectioned using an Exakt diamond band saw along 3 parallel antero-posterior planes.

### Statistical analysis

Statistical analysis was performed using Statistical Package for Social Sciences (IBM Corp, Released 2012, IBM SPSS Statistics for Windows, Version 21.0, Armonk, NY). Variables were presented as median and interquartile range (25th–75th percentile). Preoperative and postoperative continuous variables (Table 3) were compared using the Wilcoxon’s signed rank test.

**Table 3:** Preoperative versus 3 months postoperative haematological values expressed as median and interquartile range (25th–75th)

Haematological parameters	Preoperative values	3-Month values	*P*-Value
Haemoglobin (g/dl)	13.6 (13.2–13.9)	13.5 (13–13.7)	NS
Haematocrit (%)	39.9 (39.2–40.4)	36 (32.2–37.5)	NS
LDH (U/l)	762 (749.7–781.7)	781 (725.5–938.5)	NS
Bilirubin (mg/dl)	0.3 (0.2–0.3)	0.08 (0.03–0.1)	NS
K+ (mmol/l)	6 (5.7–6.1)	4.6 (4.2–5)	NS

Median (25th–75th): median and interquartile range (25th–75th percentile).

LDH: lactic dehydrogenase; NS: not significant.

## RESULTS

For chronic *in vivo* testing, devices were implanted in 10 Asaf female sheep with 94 (87.5–97) kg of body weight. Seven of the 10 sheep completed the full 3 months of observation time. Two sheep died because of severe bleeding at the cannulation site. Only one device-related death has been observed. Namely, 1 sheep has been suppressed after 28 days following severe MR detected by auscultation first and confirmed by echocardiogram due to infective endocarditis. The gross evaluation showed clear vegetations on the mitral device and leaflets as for endocarditis. Among the 3 dead animals, only 1 presented preoperative MR. Intraoperatively, in all animals, the device was successfully and easily implanted in the mitral valve annulus with the 2 legs and the connecting anterior bridge crossing through the valve commissures in all animals. In the last 4 sheep, where native chordae were torn and replaced by the artificial ones, the estimated chordal length was correct in all cases and no adjustments were required. Intraoperative data are reported in Table 1.

During all 3 months of follow-up controls, echocardiograms confirmed that left ventricular ejection fraction was normal with no significant difference with preoperative values [60 (58.7–65)% vs 60 (60–65)%, *P* = 0.1]. Anterior and posterior mitral leaflet movements were free, and MR was absent in all instances. Transvalvular peak pressure gradient was 9 (7.5–10) mmHg, and the mean gradient was 4 (3.5–4) mmHg. In summary, echocardiography showed excellent MV function in all animals at 3 months of follow-up (Video 1). Haematological studies showed no haemolysis, as evidenced by an increased Hb (from 10.1 ± 1.6 to 13 ± 0.2 g/dl) and Hct (from 28.8 ± 4.1% to 36.6 ± 4%) from early postoperative values back to values similar to those of preoperative values at 3 months of follow-up. Bilirubin and LDH level remained within normal limits, with no significant difference compared to preoperative values (Table 3).

**Figure 3:**
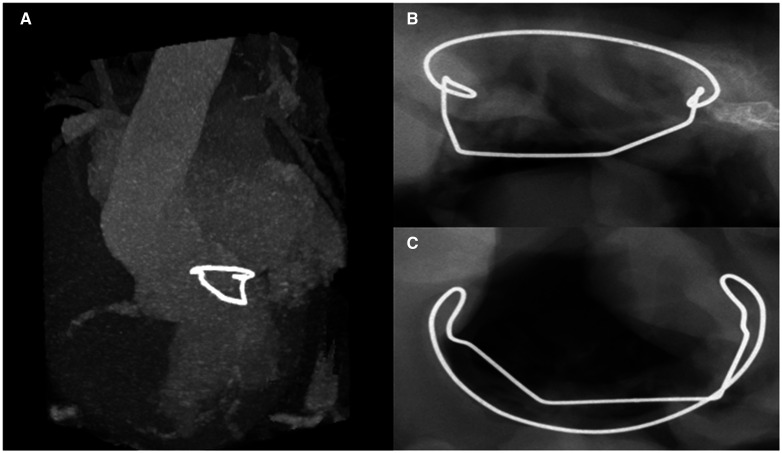
*In vivo* 3-month computed tomography scan of the animal model showing the preserved structure and the normal positioning of the device (**A**). Postoperative heart explantation X-ray in coronal lateral (**A**) and axial (**B**) views.

A computed tomography scan of 1 sheep was performed, showing the correct positioning of the device in the LV at three-dimensional reconstruction (Fig. 3 and Video 2). Following euthanasia and heart explantation, an X-ray examination was also performed, which detected no fractures. The surrounding tissue showed no evidence of calcification (Fig. 3).

**Figure 4:**
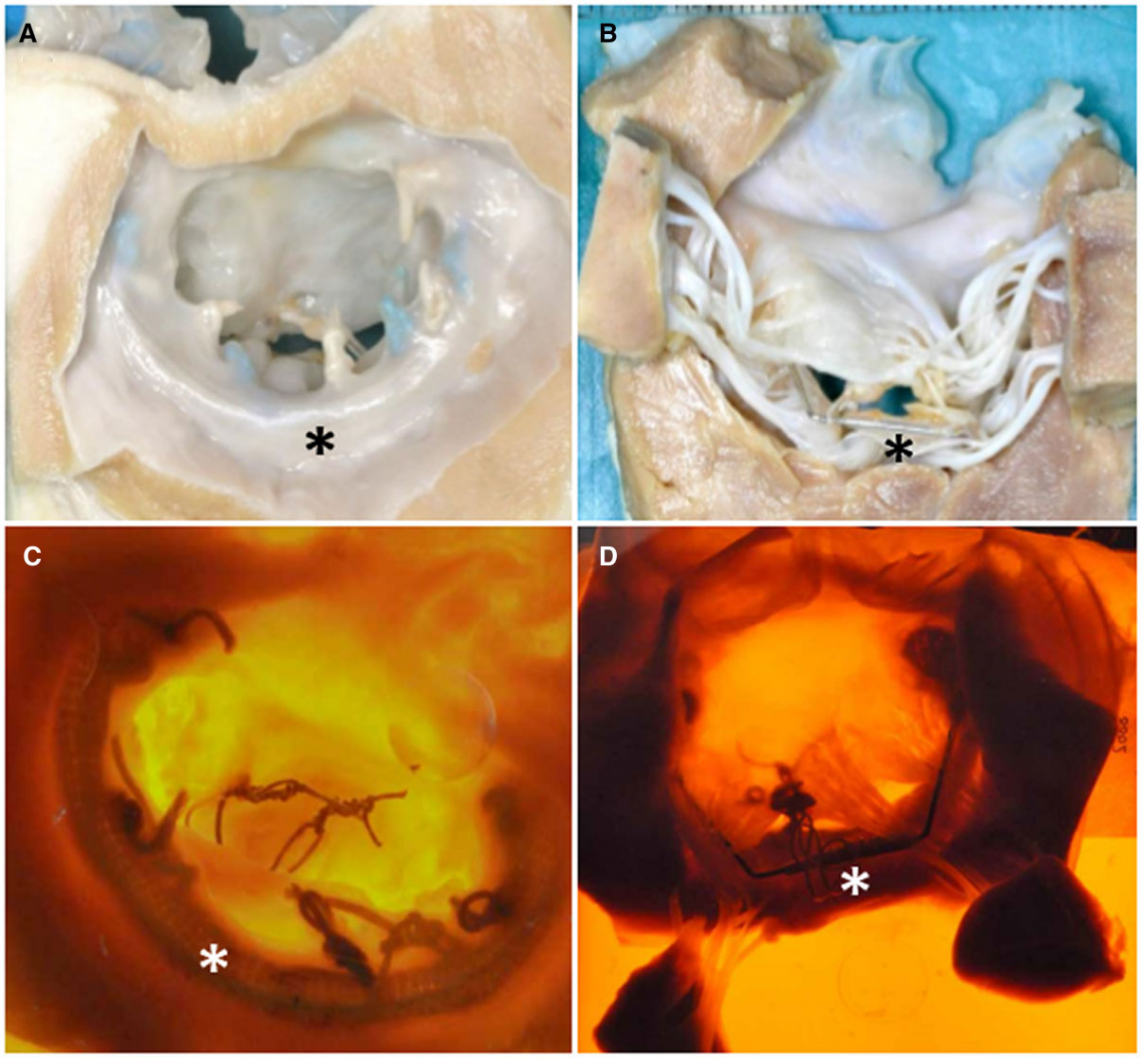
Autopsy specimen of the device implanted (asterisks) with digital block images. Atrial view (**A** and **C**) and ventricular view (**B** and **D**).

**Figure 5:**
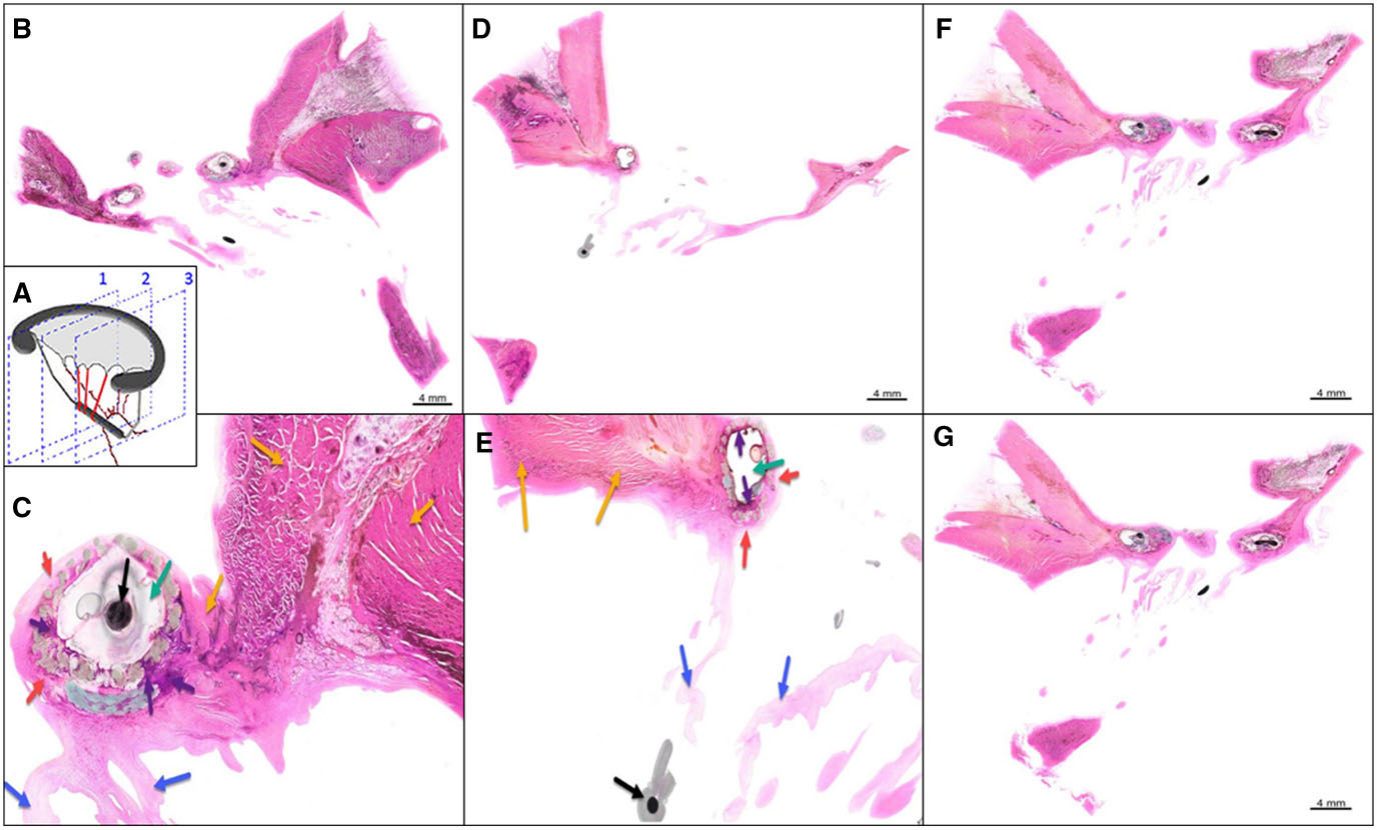
Histopathological evaluation showing normal fibroelastic and muscular tissues and sections of the metallic device. Three vertical plane sections (**A**). Plane 1 (**B** and **C**); plane 2 (**D** and **E**); and plane 3 (**F** and **G**). Red arrows: fibrosis; green arrows: ring location, covered with fabric; purple arrows: foreign body reaction; yellow arrows: myocardium; blue arrows: leaflet and chordae tendineae; and black arrow: metal core and device legs.

At necropsy, in all cases, the device proved to be solidly fixed to the anatomic mitral valve annulus, with no macroscopic damage to the surrounding tissues, to valve leaflets, chordae and papillary muscles, which appeared normal. The annuloplasty ring was properly apposed along the atrial aspect of the native annulus and was covered by smooth and glistening tissue consistent with mature fibrocellular neointima (Fig. 4). No thrombus deposition was observed. The ventricular surface of the mitral valve showed normal leaflet conformation and apposition. Papillary muscles presented no macroscopic damage. Polytetrafluoroethylene chordae were intact and connecting the bridge to the anterior leaflet. Photomicrographs of the histological slides of the mitral valve apparatus showed normal fibroelastic and muscular tissues and sections of the metallic device (Fig. 5). No histopathology tissue reaction was observed in relation to the presence of the legs of the device.

## DISCUSSION

Since its introduction, the use of artificial Gore-Tex chordae was greatly favoured among cardiac surgeons, especially to maintain the concept of respect rather than resect [17, 18]. Several studies could demonstrate favourable results pertaining to long-term repair follow-up when chordae were implanted [19, 20]. In a study by David *et al.* study, the use of artificial chordae was found to be a very important factor in making MVR feasible. Therefore, in such cases, perfect chordal implant appears of paramount relevance in obtaining valid and stable long-term results [21, 22].

A wide variety of technical solutions has been proposed for MVR using artificial chordae, mostly for properly measuring their length and anchoring them to the delicate papillary muscles. However, to date, the correct chordal implant relied essentially on the surgeon’s skill and is considered as the Achille’s heel of the procedure [15, 17–19].

Difficulty in MVR increases when the anterior leaflet is prolapsed, resulting in a high replacement rate and when repaired characterized by a higher recurrence of regurgitation than that of the posterior one. The repair of MV regurgitation involving the anterior leaflet is more demanding and the procedure is usually characterized by the longer aortic cross-clamping time [23]. Using our device, we found that the procedure was extremely simplified and with short operative time. As already described, our device differs from all presently commercialized mitral valve rings being characterized by an intraventricular structure with 2 legs interconnected by a bridge, besides the ring component [14]. The well-known distance from the bridge to the ring enabled perfect measurement of the chordae. In this study, the pre-anchoring chordal length estimation was correct, resulting in excellent repair and short operative time. Although all procedures were performed by 1 surgeon, still, the device might simplify complex procedures without requiring a long learning curve.

The device used in the present study is made of cobalt chromo, a material used also for other mitral valve rings because compared to nitinol, it does not require moulding and has no memory shape. While searching for the best configuration of the intraventricular segment of the device, we had to frequently modify its geometric form.

One of the most important issues, considering the MV apparatus including leaflets and chordae and the risk of device valve interference and damage, is the requirement of an optimal geometric form. In comparison with the applied cobalt chrome, the better performance and property in terms of fatigue of the nitinol, likely it will be the material of choice once the design is frozen. Furthermore, because of its shape memory properties, the nitinol frame may be suitable for future possible minimally invasive or percutaneous transcatheter procedures.

To evaluate the impact of the device itself on the valve apparatus, we followed the same logic of device implantation solely without chordae implantation. Therefore, considering that the device is of a three-dimensional configuration, we also used a one-sized device (24 mm); further evaluation of devices with different sizes should be performed.

Negative left ventricular remodelling after MVR is the most frequent cause of recurrent late valve insufficiency, as it may alter the anatomy of the subvalvular apparatus and lead to strain and dislodgement of artificial chordae implanted on the papillary muscles, thus leading to valve incompetence. In the proposed device, neo-chordae are not anchored on the left ventricular structures but to the fixed structure of the ring bridge and the left ventricular remodelling cannot influence chordae strain and position.

Surgical chordal reconstruction is also greatly facilitated by the ease of precise chordal length measurement prior to anchoring, thus standardizing the reconstruction procedure in a short operative time. The mean time required for a chordal implantation did not exceed 3 min. Although the chordae length is established preoperatively, still it can be modified while operating if required.

Based on the leaflet to treat, posterior or anterior, the artificial chordae will be first implanted on the bridge. The length of the neochordae is calculated from the bridge to the ring plane. A perpendicular vector (posterior leaflet) or an oblique vector (anterior leaflet) will be measured. After device implantation, if an excess of tissue is observed, it would be possible to reduce the chordal length. In the case of an even more copious excess of tissue, there is the possibility of fixing the posterior leaflet directly to the bridge, without the interposition of artificial chordae, quite away from the outflow tract, reducing the posterior leaflet height, thus likely eliminating the risk of systolic anterior motion. The anterior leaflet would then widely coapt with the upper surface of the posterior leaflet. However, the interventricular bridge, being a fixed structure without the elastic properties of the papillary muscles, might lead to greater tension on the neochordae, with a possible higher risk of chordal rupture. We implanted 5–0 chordae, and after 3 months of follow-up, we did not observe any cases of chordal rupture. However, to further reduce this risk, artificial chordae with a greater thickness (for example 4–0), could also be used.

This simple way of performing an MVR would help with standardizing the surgical procedure and might increase the repair rate.

### Limitations

We recognize that our study presents some limitations related to the relatively limited number of animals tested and the relatively short time of observation, which does not provide definitive information about long-term durability of the device.

Out of 10 sheep, 3 died (for postoperative bleeding or infective endocarditis) and only a minor portion of animals had preoperative MR. Furthermore, as this study was an experimental analysis, the lack of experience in human beings also represents a significant limitation. We treated normal valves with normal LV volumes, thereby not respecting the real pathological conditions. Meanwhile, the lack of excessive leaflet tissue, common in chronic degenerative regurgitation, did not leave margins of error. Despite the encouraging echocardiographic data, only epicardial and transthoracic echocardiogram were performed with the related limitations in acquiring the conventional parameters in sheep. Furthermore, because of the anatomical characteristics of sheep, transoesophageal echocardiogram was not feasible.

## CONCLUSIONS

In conclusion, despite limitations, this chronic *in vivo* experiment would confirm the possible safety, efficacy and potential superiority over the current benchmark surgical techniques. In addition to the ring component, the interventricular bridge could represent an additional useful tool to simplify the neochordae implantation procedure and eventually increase the MVR rate especially if anterior leaflet correction is required, by avoiding manipulation of the delicate and usually poorly exposed papillary muscle, correct chordae length important in particular when several chordate are applied and shortening the procedural time. A cautious, controlled clinical use in human beings would be expected.

## Notes

Presented at the 35th Annual Meeting of the European Association for Cardio-Thoracic Surgery, Barcelona, Spain, 13–16 October 2021.

## ABBREVIATIONS

CPB: Cardiopulmonary bypass
Hb: Haemoglobin
Hct: Haematocrit
LDH: Lactic dehydrogenase
LV: Left ventricle
MR: Mitral regurgitation
MVR: Mitral valve repair
